# COVID-19: The daunting experience of healthcare workers in Sardinia, Italy

**DOI:** 10.1017/ice.2020.149

**Published:** 2020-04-20

**Authors:** Saverio Bellizzi, Maura Fiamma, Luigi Arru, Gabriele Farina, Antonio Manca

**Affiliations:** 1 Independent Medical Epidemiology Consultant, Geneva, Switzerland; 2 Polo Ospedaliero San Francesco, ASSLL, Nuoro, Italy; 3 Independent Neurology Consultant, Sassari, Italy; 4 Candiolo Cancer Institute FPO, IRCCS, Italy

*To the Editor—*SARS-CoV-2, like other emerging high-threat pathogens, has infected health-care workers (HCWs) in China and several other countries. However, where the infection prevention and control strategies were strictly taken, nosocomial transmission has not been a major amplifier of transmission in this pandemic.^1^ As of early March, the National Health Commission of China indicated that >3,300 HCWs had been infected (of whom 22 died).^2^

In Italy, HCW infections make up 9.0% of Italy’s COVID-19 cases,^3^ which represents a serious concern because HCWs who are infected, if identified by a proper test, must stay away from healthcare facilities for at least 14 days, depleting the already exhausted workforce.

Major disparities exist across regions in Italy: by the end of the third epidemiological week of March 2020, of the 176 notified COVID-19–positive patients in Sardinia, 69 (39.2%) were HCWs,^4^ which is a striking finding when compared with the national average. In the same period, the largely hit Lombardy region recorded 3,957 affected HCWs of a total 28,750 cases (13.8%).^4^

During the fourth epidemiological week of March, the 3 major Labor Unions (CGIL, CISL, and UIL) suggested that the percentage of HCWs infected was ~50.0% of the total. Proportions soared in the province of Sassari, where 6 of 10 new cases were HCWs.^5^ On the other hand, the most recent biweekly national epidemiological report indicates that 200 HCWs of 490 total persons tested in Sardinia were positive for SARS-CoV-2.^6^

Such figures refer to something of unique relevance at the international level during this pandemic, and they align with findings from small outbreaks in Marburg and from Ebola virus outbreaks in which the nonendemic disease was suddenly introduced by an isolated traveler or individual.^7^

Sassari is one of the few European settings that witnessed a case of Ebola during the large 2014 West African epidemic.^8^ This event led to the implementation of a preparedness plan in line with international standards, with great emphasis on the use of PPE and appropriate protocols for the protection of health personnel.

Conversely, during this COVID-19 epidemic, PPE shortages have been described in several affected facilities, and some medical staff are waiting for equipment while already seeing patients who may be infected or are supplied with equipment that might not meet requirements.

Sardinia island has some geographical and demographical peculiarities that should have been taken into account. Even if it is the second largest island in Mediterranean sea and the third largest Italian region by surface ranking (ie, bigger than Lombardy), it has 1.66 million inhabitants with a population density of 69 inhabitants per square kilometer, which that is the third lowest in Italy and far lower than that of Lombardy (ie, 422 inhabitants per square kilometer). Insularity could be an advantage during the pandemic, but notably, the current global epidemic started in late December 2019, which should have provided ample time to prepare well.

Also, many people from northern Italy moved to the island, filling up holiday houses before the government’s resolution to reduce and control air and naval transportation. Only 11,000 people reported for quarantine, and the number of unreported immigrants is unknown.^9^ Such a large number of people coming from a “red zone,” could have led to a sudden increase in COVID-19 cases.

Given the ongoing severe situation in Lombardy, with >300 deaths per day in 27 April 2020, a similar trend coupled with nosocomial spread of SARS-CoV-2 among HCWs in Sardinia might have huge consequences for the local population. Physical and mental exhaustion, the pain of losing patients and colleagues, the fear of passing the infection to their families, and the torment of difficult triage decisions have intensified a very difficult situation.

Preparedness for the ongoing coronavirus disease calls for setting up of adequately equipped health facilities while protecting HCWs, which are every country’s most valuable resource. Practical measures, such as adequate provision of PPE, protocols, and continuous programs of education and training in PPE donning and doffing, should be considered a priority to ensure the safety of HCWs in current and future outbreaks.

New infections continue to emerge, and this constant threat urgently requires a cultural shift toward preparedness.
